# 硼替佐米、糖皮质激素为基础的联合方案治疗6例复发/难治免疫性血栓性血小板减少性紫癜

**DOI:** 10.3760/cma.j.issn.0253-2727.2023.05.010

**Published:** 2023-05

**Authors:** 杰 殷, 竑 田, 丹青 孔, 云 李, 骋圆 顾, 德沛 吴, 自强 余

**Affiliations:** 苏州大学附属第一医院血液内科，国家血液系统疾病临床医学研究中心，江苏省血液研究所，国家卫生健康委员会血栓与止血重点实验室，血液学协同创新中心，苏州 215006 Department of Hematology, The First Affiliated Hospital of Soochow University, National Clinical Research Center for Hematologic Diseases, Jiangsu Institute of Hematology, NHC Key Laboratory of Thrombosis and Hemostasis, Collaborative Innovation Center of Hematology, Soochow University, Suzhou 215006, China

**Keywords:** 硼替佐米, 联合治疗, 血栓性血小板减少性紫癜, Bortezomib, Combination therapy, Thrombotic thrombocytopenic purpura

## Abstract

**目的:**

观察硼替佐米联合糖皮质激素为基础的联合治疗方案在复发/难治免疫性血栓性血小板减少性紫癜（iTTP）中的疗效和不良反应。

**方法:**

纳入6例复发/难治iTTP患者，采用硼替佐米联合糖皮质激素为基础的联合方案治疗。观察患者临床缓解情况、ADAMTS13活性/ADAMTS13抑制物变化及治疗相关不良反应的发生情况。

**结果:**

6例iTTP患者中，男2例，女4例，中位年龄为21.5（18～68）岁，难治性1例，复发性5例。糖皮质激素参照泼尼松1 mg·kg^−1^·d^−1^等效剂量给药，获得临床缓解后逐渐减量；硼替佐米1.3 mg/m^2^皮下注射，第1、4、8、11天，28 d一个疗程，共2个疗程。6例iTTP患者均获得临床缓解，ADMATS13活性恢复正常，ADAMTS13抑制物转为阴性。不良反应包括血小板减少、末梢神经炎和腹痛，均恢复正常。中位随访26（9～41）个月，5例患者持续缓解，1例患者16个月后复发。

**结论:**

以硼替佐米硼替佐米联合糖皮质激素为基础的联合治疗对复发/难治iTTP具有较满意的疗效且不良反应可控。

## Abstract (en)

**Objective:**

To observe the efficacy and adverse reactions of a combination therapy regimen based on bortezomib and glucocorticoids in recurrent/refractory immune thrombocytopenic purpura（iTTP）.

**Methods:**

Six patients with recurrent/refractory TTP were included and treated with a glucocorticoid and two courses of bortezomib-based regimen. The clinical remission status of patients, changes in ADAMTS13 activity/ADAMTS13 inhibitor, and the occurrence of treatment-related adverse reactions were observed.

**Results:**

Of the 6 patients, 2 were males and 4 were females, with a median age of 21.5（18–68）years. Refractory TTP was found in 1 case and recurrent TTP in 5 cases. Glucocorticoids were administered with reference to prednisone at 1 mg·kg^−1^·d^−1^, and gradually reduced in dosage after achieving clinical remission. Bortezomib is subcutaneously administered at 1.3 mg/m^2^ on days 1, 4, 8, and 11 with a 28-day treatment course consisting of 2 courses. Six patients achieved clinical remission after receiving bortezomib as the main treatment. ADMATS13 activity returned to normal in all patients with TTP after treatment, and the ADAMTS13 inhibitor turned negative. Thrombocytopenia is the most common adverse reaction after treatment, with other adverse reactions, including peripheral neuritis and abdominal pain, but ultimately all patients returned to normal. In a median follow-up of 26（9–41）months, 5 patients maintained sustained remission, and 1 patient relapsed after 16 months of bortezomib treatment.

**Conclusion:**

Combination therapy of bortezomib and glucocorticoids has a satisfactory therapeutic effect and controllable adverse reactions for recurrent/refractory iTTP.

血栓性血小板减少性紫癜（TTP）是一种罕见而严重的血栓性微血管病，其临床特征包括微血管病性溶血性贫血、消耗性血小板减少和终末器官功能障碍。其主要发病机制是各种原因导致血管性血友病因子（VWF）裂解酶ADAMTS13失活。TTP分为先天性TTP（由ADMATS13基因突变引起）和免疫介导的TTP（iTTP）。后者与ADAMTS13自身抗体的产生有关，这些抗体通过中和ADMATS13酶活性或加速血浆ADMATS13的代谢而致病[1]。

在最新的国际血栓和止血学会（ISTH）指南[2]和国内指南[3]中，iTTP的一线治疗包括糖皮质激素、血浆置换、利妥昔单抗和卡普赛珠单抗。然而，对于难治和复发性iTTP（尤其是利妥昔单抗治疗后复发的患者）目前尚无推荐治疗方案。N-乙酰半胱氨酸、环孢素A、霉酚酸酯、环磷酰胺、脾切除术都曾用于治疗这类患者，但疗效均不理想且存在一些不良反应。2013年，Shortt等[4]首次使用蛋白酶体抑制剂硼替佐米治疗1例难治性TTP并获得成功。硼替佐米常用于多发性骨髓瘤的联合化疗，其不良反应有血小板减少、周围神经病变、心脏和胃肠道毒性以及病毒感染等[5]–[6]。我们采用硼替佐米联合糖皮质激素为基础的联合方案治疗6例难治/复发性iTTP患者并获得较好疗效。

## 病例与方法

一、病例及诊断

本研究分析了自2017年10月1日至2022年1月31日在我院接受糖皮质激素联合硼替佐米治疗的6例难治/复发性iTTP患者。所有患者均符合文献[3]的诊断标准，ADAMTS13抑制物均为阳性：①复发性TTP：在获得临床缓解后，再次出现血小板计数<100×10^9^/L且ADAMTS13活性<10％，伴或不伴有器官缺血损伤临床证据[3]。②难治性TTP：进行5次血浆置换和糖皮质激素治疗后，血小板计数仍持续减少或血小板计数<50×10^9^/L，且乳酸脱氢酶（LDH）持续升高（>1.5倍的参考值上限）[7]。DAMTS13活性测定采用改良的残余胶原结合实验，患者血浆与正常混合血浆37 °C孵育3 h以1∶9混合后ADAMTS13残留活性低于10％，则判定为ADAMTS13抑制物阳性[8]。

二、治疗方案

所有患者均采用硼替佐米联合糖皮质激素为基础的治疗方案。糖皮质激素参照等效剂量泼尼松1 mg·kg^−1^·d^−1^给药，达到临床缓解后逐渐减量。硼替佐米1.3 mg/m^2^皮下注射，第1、4、8、11天各1次，28 d为一个疗程，共2个疗程。4例患者进行了血浆置换，4例抗核抗体阳性患者加用羟氯喹200 mg每日2次。6例患者中4例既往用过利妥昔单抗。

三、疗效评估和随访

疗效评估标准参照血栓性血小板减少性紫癜诊断与治疗中国指南（2022年版）[3]。通过查阅患者住院或门诊病历随访及电话随访。末次随访时间为2022年7月31日。

## 结果

一、临床特征

本组6例患者中，男2例，女4例，中位年龄为21.5（18～68）岁，难治性TTP 1例，复发性TTP 5例。患者具体临床资料见表1。例4病史长达9年，本次是第9次复发，是复发次数最多的患者。例2第5次复发，复发时处于妊娠34周+5天，该患者经过糖皮质激素联合血浆置换治疗后顺利分娩1男婴，分娩后加用硼替佐米治疗。6例患者均伴有其他疾病，最常见的是感染，结缔组织病次之。

**表1:** 6例复发/难治免疫性血栓性血小板减少性紫癜患者硼替佐米联合糖皮质激素治疗前临床及实验室检测结果

例号	年龄（岁）	性别	伴随疾病	既往治疗	疾病状态	HGB（g/L）	PLT（×10^9^/L）	LDH（U/L）	肌酐（µmol/L）	其他自身抗体
1	46	女	干燥综合征，肺部感染	泼尼松，血浆置换，利妥昔单抗，N-乙酰半胱氨酸，环孢素A	难治性	85	2	3022	56.2	ANA 1:320；anti-nRNP（+++）；anti-SSA（++）
2	21	女^a^	肺部感染	泼尼松，血浆置换，环孢素A	第5次复发	51	10	1468	84.3	阴性
3	22	男	皮肤软组织感染	泼尼松，血浆置换，利妥昔单抗	第2次复发	151	35	257	52.2	阴性
4	44	男	获得性血友病A	泼尼松，血浆置换，利妥昔单抗，环孢素A	第9次复发	155	37	151	58.9	ANA 1:1000；anti-FⅧ（+）；anti-GPⅠb/Ⅸ（+）；anti-GPⅡb（+）；anti-GPIb（+）；anti-GPⅢa（+）
5	18	女	甲状腺功能减退	泼尼松，血浆置换，利妥昔单抗	第1次复发	96	69	207	23.7	ANA 1:640；anti-SSA（+）；anti-nRNP（±）；TPO-Ab（+）；Tg-Ab（+）
6	68	女	系统性红斑狼疮	泼尼松，血浆置换	第1次复发	100	3	689	61.6	ANA 1:640；anti-SSA（+）；anti-SSB（+）；anti-ds-DNA（+）；anti-histones（+）

**注** ^a^妊娠期；LDH：乳酸脱氢酶；ANA：抗核抗体；anti-nRNP：抗核糖蛋白抗体；anti-SSA：抗干燥综合征A抗体；anti-FⅧ：Ⅷ因子抗体；anti-GP：抗血小板糖蛋白抗体；TPO-Ab：甲状腺过氧化物酶抗体；Tg-Ab：甲状腺球蛋白抗体；anti-SSB：抗干燥综合征A抗体；anti-ds-DNA：抗双链DNA抗体；anti-histones：抗组蛋白抗体。6例患者治疗前ADAMTS13活性均为0，ADAMTS13抑制物均为阳性

二、实验室检查

6例患者均有血小板减少，中位PLT 22.5（2～69）×10^9^/L；4例患者有贫血，中位HGB 98（51～155）g/L；4例患者LDH升高，中位LDH 473（151～3022）U/L；中位血肌酐57.6（23.7～84.3）µmol/L。所有患者ADAMTS13活性均为0，抑制物均为阳性。4例患者还有其他自身抗体，包括抗核抗体、抗血小板膜糖蛋白抗体、甲状腺相关抗体和凝血因子Ⅷ抑制物。具体结果详见表1。

三、治疗方案和疗效评估

例1为难治性TTP，在3个多月的治疗期间先后经历了多达73次血浆置换。有2例复发性TTP患者未进行血浆置换（1例患者ADAMTS13检测结果回报时血小板已恢复正常，另1例患者因为血浆供应受限）。所有患者经过糖皮质激素联合2个疗程硼替佐米治疗后均获得临床缓解，ADAMTS13活性恢复正常，抑制物阴性。具体结果见表2。

**表2:** 6例复发/难治免疫性血栓性血小板减少性紫癜患者硼替佐米联合糖皮质激素治疗情况、不良反应及随访结果

例号	硼替佐米给药次数	血浆置换（次）	其他药物治疗	不良反应	随访时间（月）	随访截止时疾病状态
1	8	73	泼尼松，羟氯喹，N-乙酰半胱氨酸	手足麻木	41	缓解（泼尼松+羟氯喹维持）
2	8	5	泼尼松	无	37	复发（泼尼松维持）
3	8	0	泼尼松	血小板减少（最低46×10^9^/L）	23	缓解（停药）
4	8	2	泼尼松，羟氯喹	血小板减少（最低17×10^9^/L），手足麻木	29	缓解（停药）
5	8	0	泼尼松，羟氯喹	无	10	缓解（甲硫咪唑维持）
6	7	4	泼尼松，羟氯喹	血小板减少（最低37×10^9^/L），腹痛	9	缓解（泼尼松维持）

**注** 6例患者治疗后ADAMTS13活性均为100％，ADAMTS13抑制物均为阴性

四、不良反应及复发

6例患者中有3例硼替佐米治疗后出现血小板减少，例4在2个疗程的硼替佐米后出现了4级血小板减少，但临床无明显出现症状；中位血小板计数最低值为41.5（17～58）× 10^9^/L。出现治疗相关血小板减少患者，中位血小板恢复时间为27（16～49）d。2例患者（例1、例4）在硼替佐米治疗后出现1级周围神经病变（手足麻木），例4经过1个月治疗后消失，例1在3个月后康复。例6在第二个疗程硼替佐米治疗期间出现了严重腹痛，并因此取消了最后1次硼替佐米的治疗，患者腹痛在停药后3 d消失。6例患者中位随访26（9～41）个月，除1例患者（例2）复发外，其余仍处于持续缓解中。例2在硼替佐米治疗后16个月复发，使用利妥昔单抗后再次缓解，目前处于临床缓解中。随访至今，有2例患者现已停药。详见表2。

## 讨论

随着血浆置换、糖皮质激素、利妥昔单抗和卡普拉珠单抗的使用，iTTP的难治/复发率已降至10％以下[9]。近期发表一些个案报道和小系列报告显示了硼替佐米在难治/复发iTTP中的疗效[10]–[12]。2016年，Eskazan[13]总结了迄今为止最多的硼替佐米治疗iTTP病例，共包含来自7个研究的12例难治/复发性iTTP患者，其中11例获得缓解，没有发生严重的不良反应。

本研究纳入6例难治/复发性TTP患者，他们对利妥昔单抗、环孢素A耐药或治疗后反复复发。所有患者治疗后均获得临床缓解，这与既往的一些文献报道相似[10]–[13]。此外，这些患者还伴有感染、结缔组织疾病、甲状腺功能亢进、获得性血友病A或者处于妊娠期，这使得TTP更容易复发，治疗选择也更困难。其中4例患者除了有ADMATS13抑制物，还检测到其他抗体（抗核抗体、甲状腺相关抗体、血小板膜糖蛋白抗体，甚至罕见的凝血因子Ⅷ抗体），提示患者体内有严重的体液免疫紊乱，而硼替佐米似乎对此也有效。最近的研究表明，硼替佐米对于一些自身免疫疾病（系统性红斑狼疮、类风湿关节炎）也有疗效[14]。硼替佐米治疗iTTP的机制尚不完全清楚，但可能与清除残留的反应性B细胞和浆细胞，以及抑制树突细胞将ADAMTS13抗原向CD4^+^ T细胞递呈，从而减少ADAMTS13抑制物产生有关[15]。

至随访终点，本组6例患者中5例处于持续临床缓解截止。例4病史长达9年，TTP共发作10次，硼替佐米治疗后至今已随访了29个月，仍处于临床缓解状态。该患者目前血小板计数（70～90）×10^9^/L、ADAMTS13活性正常、ADAMTS13抑制物阴性，但血小板膜糖蛋白抗体阳性，诊断为免疫性血小板减少症，未予以特殊治疗。例2第5次发病时处于妊娠晚期，在血浆置换后行剖宫产，加用硼替佐米联合泼尼松和血浆置换，最终也获得临床缓解。Patriquin等[12]用硼替佐米治疗6例难治性iTTP患者，除1例在治疗过程中死亡外，其余5例患者平均随访17个月，均缓解。由于复发/难治性iTTP患者极为罕见，目前尚无硼替佐米治疗大宗病例复发/难治性iTTP长期疗效的研究数据。标准剂量利妥昔单抗治疗急性TTP 27个月后复发率为10.8％[16]。本组6例难治/复发性TTP病例硼替佐米治疗后中位随访26（9～41）个月，仅有1例复发，显示出令人满意的疗效。

本组6例iTTP患者中3例在硼替佐米治疗后出现血小板减少。由于这些患者同时进行了血浆置换，ADAMTS13活性并不能准确反映TTP状态。因此，血液科医生很难判断治疗期间的血小板减少是由于TTP疾病加重还是药物所致，可能导致血浆置换次数增加。根据我们的经验，此时需结合网织红细胞计数、破碎红细胞比例和患者的临床症状进行疾病状态评估。硼替佐米导致的血小板减少在多发性骨髓瘤患者中很常见，但既往治疗TTP的文献中未见报道[10]–[13]。可能的原因是硼替佐米多用于难治/复发性TTP患者，其引起的血小板减少与疾病导致的血小板减少相重叠。此外，有些iTTP患者在血小板恢复后未再接受第2个疗程硼替佐米治疗。

除血小板减少外，本组iTTP患者还出现了周围神经病变和腹痛。但在随访过程中均恢复了正常。虽然有硼替佐米治疗TTP患者诱发感染的报道[17]，但本组患者没有出现严重感染。此外，还有学者报告硼替佐米治疗多发性骨髓瘤诱发TTP或其他血栓性微血管病[18]–[19]。

本组难治/复发性iTTP患者除了使用硼替佐米和糖皮质激素外，多数患者还进行了血浆置换，在客观评价硼替佐米的疗效时不能忽视其他治疗措施的作用。在既往文献报道中，1例继发于卡波氏肉瘤的复发/难治性iTTP患者对包括硼替佐米在内多种治疗措施无效并最终死亡[20]，另有1例复发/难治性iTTP患者合并克隆恩病[21]，这也提示当TTP继发于其他疾病时，更容易复发或变得难治。最新的报道显示一种浆细胞靶向药物达雷妥尤单抗治疗2例复发性iTTP获得成功[22]，患者ADAMTS13自身抗体消失，ADAMTS13活性恢复正常。这也提示浆细胞在复发性iTTP中可能是一个重要的致病环节（尤其是利妥昔单抗治疗后复发的患者），值得进一步研究。

综上所述，以硼替佐米、糖皮质激素为基础的联合治疗方案在本组6例难治/复发性iTTP患者中获得良好疗效，虽然产生了血小板减少、手足麻木和腹痛等不良反应，但均为可逆性，提示以硼替佐米、糖皮质激素为基础的联合治疗可作为难治/复发性iTTP患者的较好治疗选择。
